# Prospects for the Use of New Technologies to Combat Multidrug-Resistant Bacteria

**DOI:** 10.3389/fphar.2019.00692

**Published:** 2019-06-21

**Authors:** Renata Lima, Fernando Sá Del Fiol, Victor M. Balcão

**Affiliations:** ^1^LABiToN—Laboratory of Bioactivity Assessment and Toxicology of Nanomaterials, University of Sorocaba, Sorocaba, Brazil; ^2^CRIA—Antibiotic Reference and Information Center, University of Sorocaba, Sorocaba, Brazil; ^3^PhageLab—Laboratory of Biofilms and Bacteriophages, i(bs)^2^—intelligent biosensing and biomolecule stabilization research group, University of Sorocaba, Sorocaba, Brazil; ^4^Department of Biology and CESAM, University of Aveiro, Campus Universitário de Santiago, Aveiro, Portugal

**Keywords:** multidrug-resistant bacteria, bacteriophage particles, phage therapy, CRISPR–Cas, nanotechnology

## Abstract

The increasing use of antibiotics is being driven by factors such as the aging of the population, increased occurrence of infections, and greater prevalence of chronic diseases that require antimicrobial treatment. The excessive and unnecessary use of antibiotics in humans has led to the emergence of bacteria resistant to the antibiotics currently available, as well as to the selective development of other microorganisms, hence contributing to the widespread dissemination of resistance genes at the environmental level. Due to this, attempts are being made to develop new techniques to combat resistant bacteria, among them the use of strictly lytic bacteriophage particles, CRISPR–Cas, and nanotechnology. The use of these technologies, alone or in combination, is promising for solving a problem that humanity faces today and that could lead to human extinction: the domination of pathogenic bacteria resistant to artificial drugs. This prospective paper discusses the potential of bacteriophage particles, CRISPR–Cas, and nanotechnology for use in combating human (bacterial) infections.

## Bacterial Resistance

Since their discovery in 1929, antibiotics have been widely used in human and veterinary medicine, either for treatments or in attempts to prevent bacterial infections. The excessive use of antibiotics, whether for prevention or treatment, has significantly increased the level of bacterial resistance worldwide (Ali et al., 2018). The associated numbers of human deaths are alarming, reaching 50,000 per year in the United States and Europe (Simlai et al., 2016), with an estimated 10 million deaths per year by 2050, surpassing the current deaths resulting from all types of cancer (approximately 8.2 million) (Jansen et al., 2018).

The first list of antibiotic-resistant pathogens was published by the World Health Organization (WHO) in 2017. This list showed that out of the 12 resistant pathogens, seven were noted to be resistant to beta-lactam antibiotics. Consequently, there is renewed focus on the production of new antibiotics, establishing a goal for future research strategies (WHO, 2017).

The overuse and misuse of antibiotics in humans have led to the selective emergence of bacteria resistant to the currently available antibiotics, as well as resistant non-pathogenic microbiota, hence leading to the generalized dissemination of resistance genes at the environmental level (Nitsch-Osuch et al., 2016). There is greatest concern when this phenomenon occurs with *Enterococcus* spp., *Staphylococcus aureus*, *Klebsiella pneumoniae*, *Acinetobacter baumannii*, *Pseudomonas aeruginosa*, and *Enterobacter* spp., together given the acronym ESKAPE, which highlights the ability of these microorganisms to escape the action of antimicrobial agents (Boucher et al., 2009).

Antimicrobial resistance has become globalized, following the first reports of its appearance in India, with its subsequent spread to Pakistan, the United States, Canada, Japan, and the United Kingdom (Rios et al., 2016). This resistance can occur in different ways, depending on the acquired and selective genetic changes or insertion of external genes, which leads to previously non-existent responses. Several mechanisms of resistance have emerged in recent times, including alteration of the target (by a DNA gyrase), increased efflux (export of a drug out of the microorganism), inactivation of fluoroquinolones (by an aminoglycoside N-acetyltransferase), inhibition of the 30S ribosomal subunit (by aminoglycosides), and protection of the target by DNA-binding proteins (the Qnr family) (Redgrave et al., 2014; Munita and Arias, 2016; Kapoor et al., 2017).

Some of these changes are already well known, such as alteration of the chemical structure of antimicrobial agents (Alekshun and Levy, 2007), decrease of the concentration of the antimicrobial at its site of action (Gonzalez-Bello, 2017; Willers et al., 2017), changes in the target of antimicrobial action (Sieradzki and Markiewicz, 2004), and alteration of membrane permeability (Hao et al., 2018). There are mechanisms of permeability reduction that do not involve porin expression, such as changes in the cell envelope of *P. aeruginosa* that are associated with resistance to polymyxin B (Falagas and Kasiakou, 2005). In addition to antibiotics that act on the cell wall, such as penicillins and glycopeptides, the activities of other antimicrobials that act on the bacterial ribosome may also decrease due to changes in their primary target. This phenomenon mainly affects macrolides and tetracyclines (Poehlsgaard and Douthwaite, 2005; Wu et al., 2005).

The presence of these mechanisms of resistance is increasingly common in large numbers of microorganisms, due to the selective pressure exerted by antimicrobials, leading to a natural selection that results in the dominance of certain groups of resistant bacteria, with concomitant death of sensitive microorganisms (Tello et al., 2012).

In a meta-analysis carried out by Bell et al. (2014), in which 243 studies were evaluated, it was concluded that “Increased consumption of antibiotics may not only produce greater resistance at the individual patient level but may also produce greater resistance at the community, country, and regional levels, which can harm individual patients.” Another study of the same year evaluated the consumption of antibiotics worldwide between 2000 and 2010. It was found that the consumption of antibiotics increased by around 36%, with the countries of the BRICS group (Brazil, Russia, India, China, and South Africa) accounting for approximately 76% of the increase (Van Boeckel et al., 2014).

Therefore, the data reflect a worrying trend regarding the treatment of infectious diseases, since not only are these drugs being increasingly used (Van Boeckel et al., 2014), but also their use is directly proportional to the increase in resistance indicators (Bell et al., 2014). In the absence of any significant discovery of new molecules for the control of resistant microorganisms (Hogberg et al., 2010), there is an urgent need for redefining the relationship of humans with infectious diseases.

In summary, the problem faced in relation to bacterial resistance is a concern that must be urgently addressed, since functional meta-genomic studies of soil microorganisms have revealed a wide range of genetic determinants that confer resistance to antibiotics, of which only one fraction has been described in human pathogens (Forsberg et al., 2014).

Hence, there is a pressing need for a new generation of antimicrobials able to mitigate the spread of antibiotic resistance and preserve beneficial microbiota. Among the possibilities for the solution of problems related to bacterial resistance, the use of nanotechnology, CRISPR–Cas9, and therapy with bacteriophage particles can be highlighted as potential future strategies. These techniques could be employed individually to directly combat microorganisms, as well as in combination in integrated strategies.

The scientific community has indicated that there are no perspectives for any significant clinical introduction of new antimicrobials in the short term. The main recommended approach is rational use of the classical antibiotics that have been used for the past 50 years, together with techniques that enhance their activity. This may be achieved using substances that increase antibiotic activity by reducing or blocking the resistance mechanism, such as beta-lactamase, efflux pump, and quorum sensing inhibitors, as well as bacteriophages and new drug delivery systems, among other techniques (Moo et al., 2019; Mulani et al., 2019; Pham et al., 2019; Vikesland et al., 2019).

## CRISPRs

**Figure 1:**
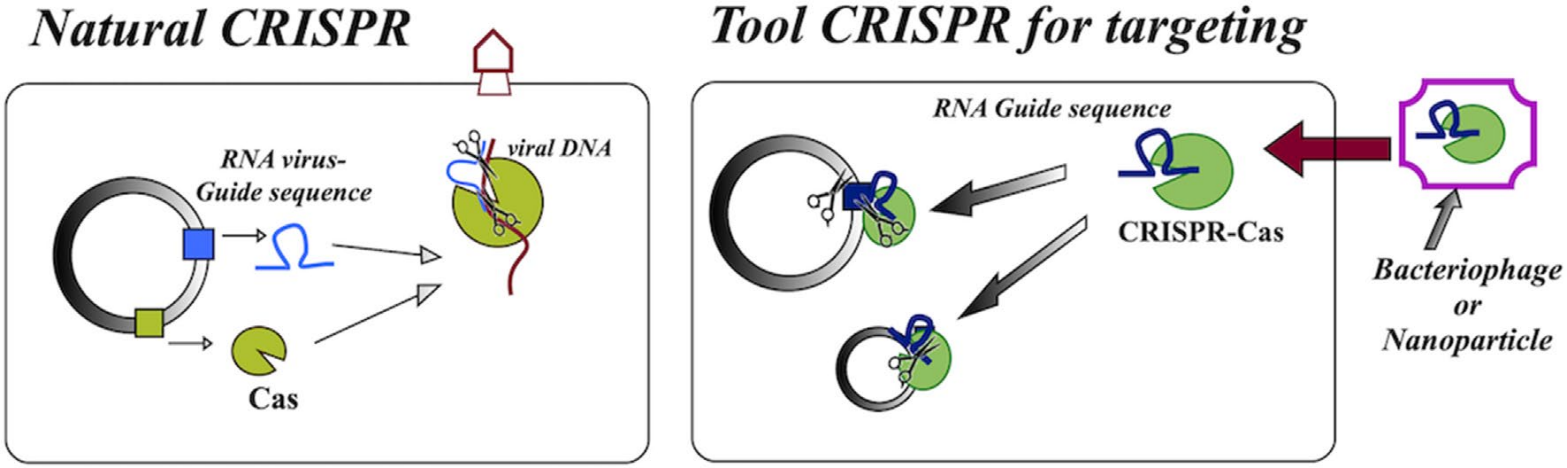
Schematic drawing showing the natural CRISPR–Cas complex found in bacteria, which functions as an “immune system” against viruses, and the CRISPR–Cas tool used as an agent, based on the complex naturally present in bacteria.

CRISPRs (clustered regularly interspaced short palindromic repeats) are adaptive immune systems derived from bacteria and archaea. CRISPR–Cas systems use RNA for target DNA recognition and the Cas enzyme for subsequent destruction of nucleic acids, so they require only one protein for binding and cleavage. Due to this simplicity, researchers have developed a new molecular tool based on natural CRISPRs (**Figure 1**). This tool has different applications, one of them being the possibility of antimicrobial action, since they are cytotoxic systems that can be directed to kill bacteria, immunizing them against resistant plasmids (Sorek et al., 2013;Bikard et al., 2014; Hsu et al., 2014).

For medical purposes, CRISPR–Cas systems can enable the selective and specific removal of microorganisms. Although there are other antimicrobial approaches, they offer only partial solutions, while CRISPR systems are generalized and programmable strategies (Gomaa et al., 2014) that can be employed to selectively and quantitatively remove individual bacterial strains, based purely on sequence information, hence creating opportunities in the treatment of multidrug-resistant infections.

In studies of the use of these systems as antimicrobials, Gomaa et al. (2014) reported that both heterologous and endogenous systems could selectively kill bacterial species and strains. It was shown that all sequences in the target genome led to cell death, suggesting that, theoretically, any genomic location could be a distinct target for antimicrobials based on CRISPRs. Another possibility would be the use of this technology for antimicrobial action using RNA-guided nucleases (RGNs), targeting specific resistance genes or undesirable polymorphisms, allowing programmable remodeling of the microbiota (Citorik et al., 2014).

In a study carried out by Fuente-Núñez and Lu (2017) concerning CRISPR–Cas constructs designed to function as precision antimicrobials, these were shown to be capable of eliminating drug-resistant microbes, with CRISPR–Cas selectively targeting genes involved in antibiotic resistance, biofilm formation, and virulence. However, although studies show that CRISPRs are effective, there are still problems to be overcome in relation to an efficient delivery vehicle, which is the next step for the implementation of CRISPR–Cas systems as antimicrobial agents (Beisel et al., 2014). Focusing on the problem of CRISPR transportation and delivery, Pan et al. (2017) were able to identify eight depolymerases in the multi-host bacteriophage K64-1, which, together with K64dep (S2-5), characterized elsewhere, gave a total of nine capsule depolymerases.

Currently, obtaining bacteriophages as carriers of CRISPRs is still a challenge. Shen et al. (2018) succeeded in obtaining positive results in studies aimed at obtaining a *Klebsiella* bacteriophage by genome alteration, which was suggested as a possibility for the use of targeted CRISPRs. One option is to use nanotechnology for the delivery of CRISPRs, which could provide surface modifications that ensure the desired specificity (Yan et al., 2015). As pointed out by Pursey et al., (2018), there is still a great deal to discover concerning the use of CRISPR–Cas in the fight against resistant bacteria, with further research especially needed in relation to its safe use.

Another concern is the possibility that bacteria could present resistance against CRISPR–Cas, since the original mechanisms are present in them. However, in a study by Chen et al. (2019), performed with multidrug-resistant *Shigella*, it was shown that the bacteria that presented resistance genes also presented a decrease in the activity of natural CRISPR–Cas.

If we consider the different possibilities of target genes for CRISPR–Cas, we can conclude that there is a need for an interdisciplinary study, where there is collaboration of researchers who study sequences, find a safe way of delivery, and evaluate the existence of resistance to technology. Different studies show that bacteria tend to store different genes, and different combinations between virulence and resistance are an alarming threat, as it suggests the feasibility of adaptability. A study carried out by Oliveira Santos et al. (2018), where they showed the possible adaptability of the KPC-2 gene to different mobile elements, is an example of the need to consider different possibilities for the application of CRISPR–Cas. Regarding the onset of carbapenem-resistant *K. pneumoniae*, recent publication showed the introduction of two new DNA editing systems. One is the plasmid pCasKP-pSGKP and the other is the plasmid system pBECKP, where both systems showed efficiency in genome editing, which will facilitate further investigations for treatment of resistance to carbapenems (Wang et al., 2018).

Although the CRISPR–Cas tool offers a new possibility of fighting multidrug bacteria, some studies show that they do not present activity in some strains, as demonstrated by Hullahalli et al. (2017, 2018) in studies with *Enterococcus faecalis* where they present a study that determines the genetic basis of phenotypes associated with CRISPR–Cas tolerance, showing the importance of having a better knowledge of the response of organisms and possible strategies for dealing with conflicts induced by the use of CRISPRS, which may lead to tolerant phenotypes to this tool. Therefore, these studies show that knowledge of the genome and the metabolic pathways of the different resistant multidrug bacteria should be investigated so that resistance problems will not occur in the future in relation to new strategies used to fight resistant bacteria.

## Nanotechnology in the Fight Against Resistant Bacteria

Nanotechnology applied to the synthesis of new antibiotics is an important approach, since the use of nanometric size materials can result in greater contact between the compound and the bacteria, with improved bioavailability, increased absorption, faster passage of the drug into the cell, and enhanced mucoadhesion. There is also the possibility of producing controlled release systems for the targeted delivery of encapsulated or surface adsorbed drugs (Zaidi et al., 2017;Jamil and Imran, 2018). One new approach is to use nanoparticles (NPs) of a metal such as silver, which can affect the bacterial respiration system, inducing the generation of reactive oxygen species (ROS). This approach could be used synergistically with antimicrobials, with effects such as inhibition and alteration of the synthesis of the cell wall, as well as its rupture (Shahverdi et al., 2007; Kumar et al., 2018).

One of the concerns regarding the use of nanoparticles is in relation to the resistance that bacteria can present to them, or the possibility of stimulating the transmission of MultiDrug-Resistant (MDR) genes. An example is provided by the work of Ansari et al. (2014), where Al_2_O_3_ nanoparticles were observed to promote the horizontal conjugative transfer of MDR genes, hence increasing the resistance to antibiotics.

The use of NPs to eliminate microorganisms can involve microbicidal or microbiostatic effects. In the latter case, the growth of bacteria is interrupted and the metabolic activities are halted, with microbial death then induced by the immune cells of the host. Nanotechnology can also solve problems related to drug solubility, since encapsulation can improve permeation through the membrane, increase circulation times, and enhance efficiency, while there is also the possibility of directing the drug towards the desired site of action in the body (Rodzinski et al., 2016).

The use of nanoparticles appears to have potential for the treatment of infectious diseases, especially considering that NPs may be able to access locations where the pathogens are present. However, there are a number of issues to be resolved, such as the scarcity of toxicity data, few existing preclinical studies, and the need for regulation (Zaidi et al., 2017).

### Polymeric Nanoparticles and Nanocrystals

The use of polymeric nanocapsules as carriers for antibiotics, or the use of drug nanocrystals that are stable during delivery, can be successfully applied to a range of commonly used drugs. Polylactide-*co*-glycolide (PLGA) is an especially useful substance that can be employed in nanotechnological drug delivery applications (Kalhapure et al., 2014; Hemeg, 2017;Boya et al., 2017; Shaaban et al., 2017).

Hong et al. (2017) used bacitracin A (BA) modified with PLGA for synthesis of nano-BA, resulting in a core–shell structure with an average diameter of 150 nm. It was found that the nanoparticles strongly increased the antibacterial activity, than does free BA, with effective inhibition of the growth of various types of Gram (+) and Gram (−) bacteria. The formulation provided improved wound healing in rats than did use of a commercial Polysporin^®^ ointment.

Yu et al. (2016) reported the development of a multifunctional release system with encapsulation of gentamicin sulfate/zirconium bis(monohydrogen orthophosphate) (α-ZrP) using chitosan (CHI). The formulation (α-ZrP CHI) extended the release of the drug, than did unencapsulated α-ZrP, which was attributed to the unique lamellar structure and the CHI encapsulation. The methodology provided a model for the future development of new delivery vehicles.

Shaaban et al. (2017) reported that nanoantibiotics produced by incorporating imipenem in PLGA or PCL nanocapsules provided better results, than did classical imipenem. The nanoencapsulated formulations showed antimicrobial and anti-adherent activities in evaluations using clinical isolates of imipenem-resistant bacteria.

Other types of nanoparticles that have received attention are lipid nanoparticles (liposomes) (Derbali et al., 2019) and nanoceramics applied in orthopedic surgeries where systemic drug administration has limitations (Kumar and Madhumathi, 2016).

Gaspar et al. (2017) reported the use of solid lipid nanoparticles containing rifabutin (RFB) for pulmonary administration to treat tuberculosis. The nanoparticles increased the activity of the drug against *M. tuberculosis* infection, suggesting that RFB-solid lipid nanoparticles (SLN) encapsulation could be a promising approach for tuberculosis treatment. A major advantage of encapsulation is that it provides sustained release of the drug, resulting in greater efficiency of treatment, as well as easier absorption, enabling satisfactory results to be achieved with a smaller amount of the active agent.

Although the use of nanoparticles can be advantageous, some studies have shown that the microenvironment where they are released (such as blood and lung fluid) may alter the creation of the nanoparticle–pathogen complex, due to the formation of a corona around the nanoparticle. Siemer et al. (2019) exposed nanoparticles to different bacteria and showed that formation of the pathogen–nanoparticle complex was assisted by its small size and that the presence of a corona significantly inhibited formation of the complex. Therefore, in addition to *in vitro* analyses, new studies are needed that consider the microenvironment in which the nanoparticle will be released and exert its action.

### Metallic Nanoparticles

The use of metallic nanoparticles can be a good option in the fight against resistant bacteria. Studies have reported the synthesis and use of different nanoparticulate metals, metal oxides, metal halides, and bimetallic materials showing antimicrobial activity. Nanoparticles have been synthesized consisting of Ag, Au, Zn, Cu, Ti, and Mg, among other metals (Zakharova et al., 2015; Hajipour et al., 2012;Sunitha et al., 2013; Dizaj et al., 2014; He et al., 2016; Senarathna et al., 2017;Eymard-Vernain et al., 2018). However, consideration should be given to their potential toxicity (Lima et al., 2012; Dakal et al., 2016; Durán et al., 2016a).

Eymard-Vernain et al. (2018) showed that MgO nanoparticles presented bactericidal action, mainly affecting the expression of genes related to oxidative stress, together with membrane alteration. Verma et al. (2018) reported excellent antibacterial activity of ZnO nanoparticles, with a size-dependent effect, since the use of smaller nanoparticles resulted in more ROS and increased cell membrane rupture.

Other studies have investigated the bactericidal potential of carbon nanotubes, either plain or functionalized, as well as their use to assist the transport and translocation of antibiotics (Cong et al., 2016; Mocan et al., 2017).

With the development of nanotechnology, many studies have been carried out concerning the application of nanoparticles as antimicrobials. These nanomaterials present different diameters, structures, and modes of action. Some of them have produced good results, showing that nanotechnology can be used as one of the strategies in the fight against multidrug-resistant bacteria in the future (**Supplementary Table 1**).

Silver nanoparticles are the most studied metallic nanoparticles, with their antimicrobial activity having been recognized by the United States Food and Drug Administration (FDA) since the year 1920. The mechanisms of action of silver nanoparticles (AgNP) on bacteria have been exhaustively investigated. There is a consensus that adhesion of the nanoparticles to the cell membrane can lead to electrostatic changes, porosity alteration, rupture, leakage of cytoplasmic content, interference in bacterial respiratory processes, blocking of enzyme activity, and DNA destruction. It has also been observed that there is the production of ROS, with consequent effects on the DNA (Choi and Hu, 2008; Durán et al., 2010; Prabhu and Poulose, 2012; Rai et al., 2012; Kon and Rai, 2013; Yuan et al., 2017).

The adhesion of nanoparticles to bacterial membranes mainly occurs due to the presence of proteoglycans (Kim et al., 2017) and results in rupture or increased porosity of the membrane. This enables access of the nanoparticles into the cell, where they can interact with enzymes and DNA (Grigor’eva et al., 2013; Kasithevar et al., 2017). AgNPs may also interact with membrane proteins, leading to cell stress, or may interact with the lipid part of the membrane, affecting its fluidity (Morones et al., 2005; Chwalibog et al., 2010). Some studies have suggested that the observed effects are actually caused by silver ions released from AgNPs (Jung et al., 2008; Xiu et al., 2011; Xiu et al., 2012; Chernousova and Epple, 2013). Accordingly, the AgNPs only act as vehicles for the delivery of ions that cause adverse effects in the respiratory chain and protein synthesis, as well as DNA alterations (Chen et al., 2011; Li et al., 2014).

**Figure 2:**
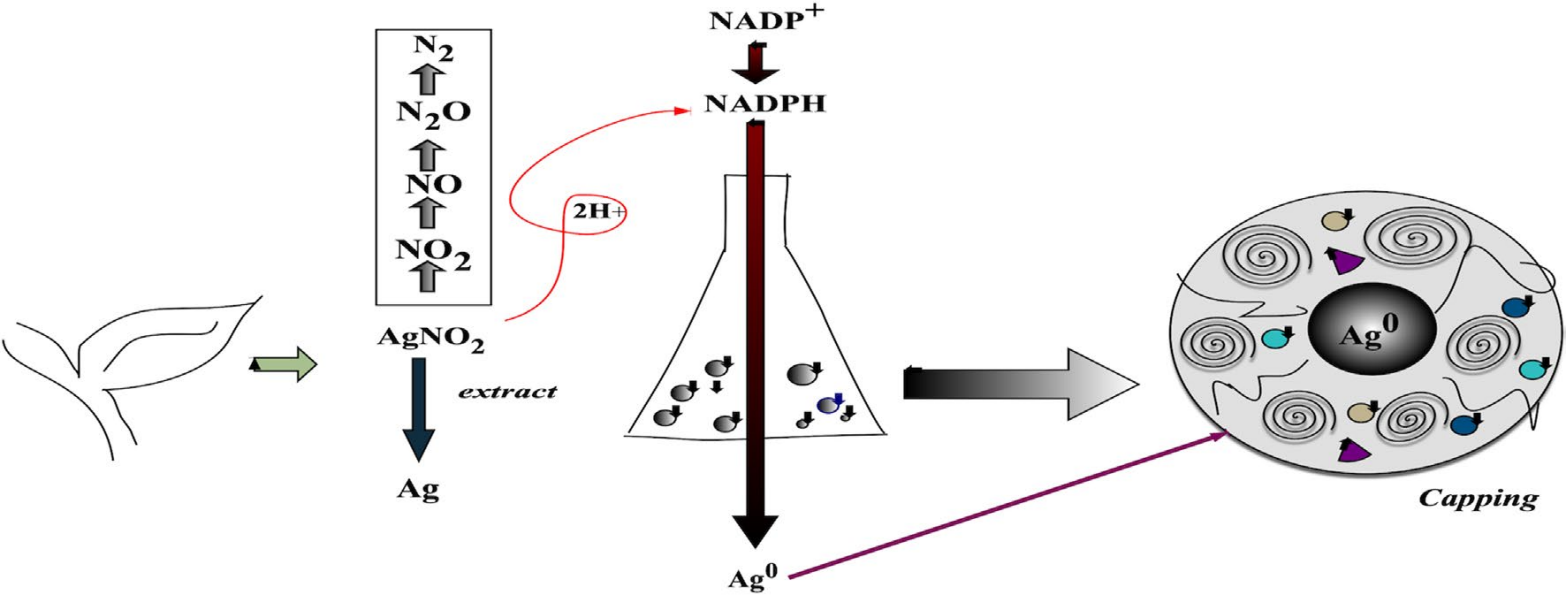
Scheme, based on the literature, illustrating the synthesis of biogenic nanoparticles. The synthesis uses AgNO_3_ together with extract (or metabolites) and enzymes from the organism. These nanoparticles have a characteristic outer layer (coating) containing metabolites.

The biogenic synthesis of silver nanoparticles (**Figure 2**) has received increasing attention in recent years. These nanoparticles present positive characteristics in terms of their improved stability and dispersion, due to the coating formed during the synthesis. In addition, there may be a positive effect of synergy between the nanoparticles and the compounds originating from the organism used. Biogenic synthesis is considered simple, low cost, and suitable for large-scale nanoparticle production (Lima et al., 2012; Kasithevar et al., 2017).

Biogenic nanoparticles have been found to present lower toxicity, while providing effective bactericidal activity against both Gram (−) and Gram (+) bacteria (Durán et al., 2016b; Kasithevar et al., 2017). These nanoparticles have also shown potential for use in the control of fungi (Balashanmugam and Kalaichelvan, 2015; Ahmad et al., 2016; Guilger et al., 2017).

### Nanocages

Nanocages are hollow and porous nanometric structures that may be used for the transport and delivery of antibiotics. They can be synthesized from various substances, including metals, proteins, and polymers, and have been investigated in terms of their potential for combating multidrug-resistant bacteria. Reported advantages of these structures are that they provide greater adhesion, retention at the site of infection, increased systemic circulation, and good biocompatibility (Wang et al., 2016; Mekeer et al., 2018).

Wang et al. (2018) synthesized gold nanocages using membrane coating of macrophages pretreated with *S. aureus*. Clinical treatments performed with local or systemic injection showed that the system provided increased bactericidal effectiveness. Ruozi et al. (2017) synthesized apoferritin-based nanocages, which were used for the encapsulation of streptomycin. The system showed promise for the delivery of antimicrobials, although further characterization, biocompatibility, and efficacy studies were still required. A study by Wu et al. (2019), using silica, silver, and gold nanospheres, showed that the Au–Ag@SiO_2_ nanocage had broad-spectrum bactericidal properties. The nanocage could be used for antibiotic transport, as well as for infrared-induced hyperthermia therapy against bacterial infection.

## Bacteriophages

Bacteriophages (or phages, for short), which are viruses that only infect bacterial cells, are among the most ubiquitous biological entities, with a total estimated abundance of at least 1,030 types (Chibani-Chennoufi et al., 2004). Despite being known for more than 100 years, only now is renewed interest in phages driving studies of them as potential alternatives or complements to current antibiotics, due to their unique affinities and ability to kill bacteria resistant to antibiotics (Hagens and Loessner, 2010; Hyman and Abedon, 2010; Summers, 2012). The interaction between phage particles and bacteria generally involves specific receptors located in the outer membranes of bacteria. Despite the great potential of phages for treating and/or controlling infections caused by antibiotic-resistant bacteria, only a few clinical trials have been performed in humans and are accepted by public health authorities such as the FDA and the European Medicines Agency (EMA) (Rios et al., 2016).

Phages are ubiquitous in the biosphere and are highly specific to particular bacteria species, acting as natural predators of bacteria. They exhibit high tissue permeability and do not affect the beneficial intestinal microflora (so they do not promote secondary infections). Their exponential growth results in their accumulation in extremely high concentrations where they are needed the most, as long as the bacterial host still exists (Hagens and Loessner, 2010; Wittebole et al., 2013; Rios et al., 2016;Harada et al., 2018). However, phage-based therapy requires that the bacterium responsible for the infection is firstly isolated, before the identification and isolation of a specific and strictly lytic phage can be achieved. In addition, due to their protein nature, plain phage particles may be recognized by the immune system, resulting in a drastic reduction of their therapeutic efficacy (Chan and Abedon, 2012; Wittebole et al., 2013).

Bacterial resistance to phage particles generally occurs due to non-adsorption, membrane coating due to mucilage production by bacteria, and destruction of viral genetic material by restriction endonucleases (Wittebole et al., 2013).

Following oral or intravenous administration, phage particles may affect the major body systems, namely, the cardiovascular, digestive, immune, and nervous systems (Moutinho et al., 2012). Furthermore, due to their protein nature, phage particles are prone to denaturation by conformational changes that may be either reversible or irreversible, or to destruction by the immune system. The solution lies in protecting them, either by encapsulation within nanocarriers (Rios et al., 2018) that are invisible towards the digestive and immune systems, or by binding them to a macroscopic support so that they become insoluble (Balcão et al., 2013; Balcão et al., 2014). The combination of these strategies can provide phages with structural and functional stabilization (Balcão and Vila, 2015), enabling them to be potentially used for the eradication of antibiotic-resistant bacteria.

Several studies have described phage-based CRISPR-driven techniques for the prevention of bacterial drug resistance (Barrangou, 2015; Bikard and Barrangou, 2017; Doss et al., 2017;Hatoum-Aslan, 2018; Pursey et al., 2018). In this approach, bacteriophages are designed to carry and deliver CRISPR–Cas in bacteria, in order to combat multidrug-resistant bacteria. Such systems are being developed by biotechnology companies such as Locus Biosciences (Morrisville, NC, USA) and Eligo Bioscience (Paris, France) (Reardon, 2017).

Recent biotechnological advances therefore open the door to the possibility of tailoring bacteriophage particles to improve their characteristics, including i) enhancing the ability of phages to penetrate bacterial biofilms; ii) increasing phage efficacy; iii) broadening the spectrum of phage lytic activities to infections caused by different bacteria; and iv) making phages more stable and specific (Maura and Debarbieux, 2011; Rios et al., 2016; Harada et al., 2018).

At the present time, due to the increase in bacterial resistance to antibiotics, together with the likely ineffectiveness of antibiotics within a few years, there is an urgent need to develop new antimicrobial strategies. This is a new era, in which the emergence of new solutions and discoveries will be crucial.

## Future Trends and Possible Solutions

**Figure 3:**
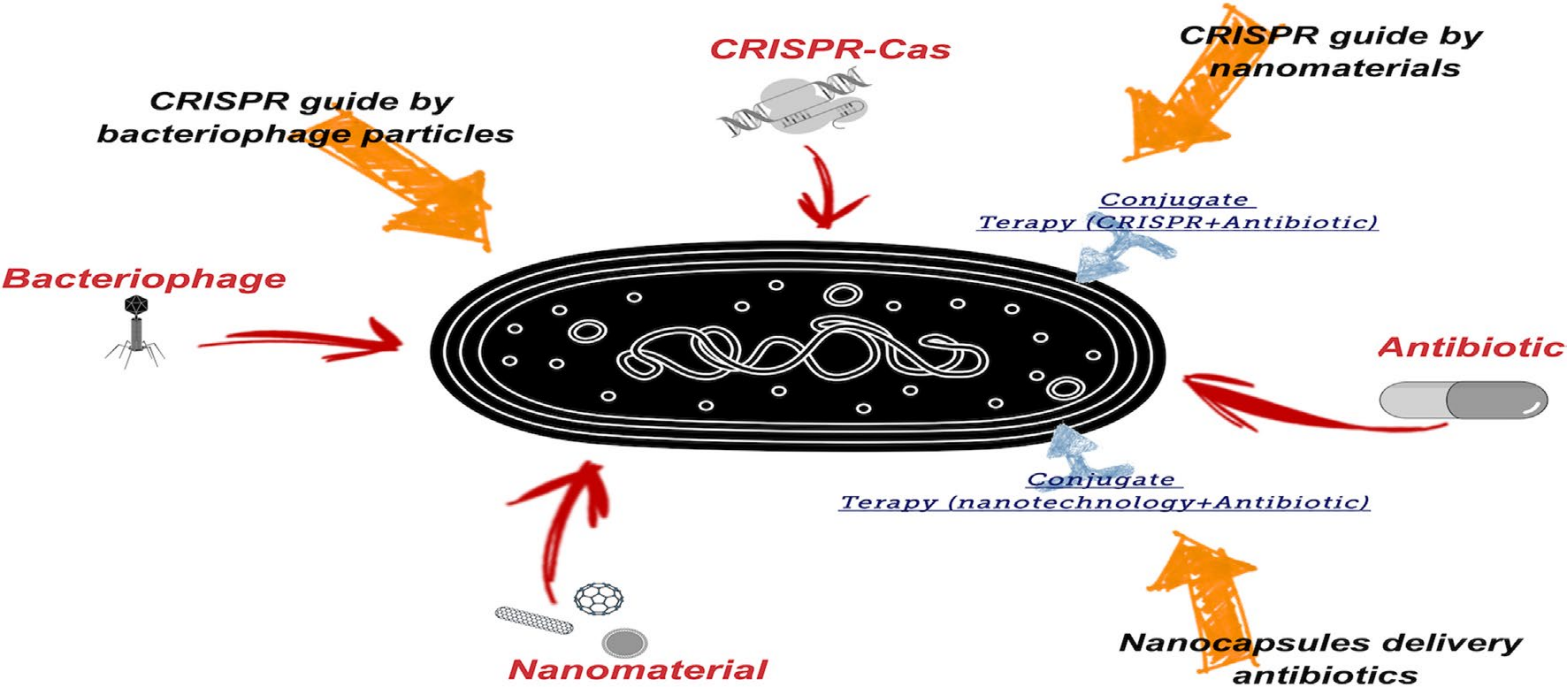
Proposed new technological tools to combat multidrug-resistant bacteria. Emphasis is given to the need to use more than one tool.

The use of new technologies to combat multidrug-resistant bacteria is ever more necessary, because although there are still effective antibiotics, resistance to them is constantly increasing. The strategies discussed in this paper may provide new ways of fighting multidrug-resistant bacteria. This could include associations between different strategies, as well as their use in combination with antibiotics, in order to combat this critical emerging problem (**Figure 3**).

The use of CRISPRs, a relatively new technology, may be one of the available solutions. Coupled with nanotechnological delivery methods, this technique could be sufficiently specific and provide the activity required to combat multidrug-resistant bacteria. For this, nanocapsules could be synthesized that are able to reach specific targets, which would facilitate the delivery of CRISPRs.

Biogenic metal nanoparticles, such as silver nanoparticles, may be an option in conjugated treatments to combat MDR bacteria. These nanoparticles offer the benefits of synergy between the effects of the metal and the metabolites of the organism used for their production. They present low toxicity and can act to disrupt existing mechanisms of resistance in bacteria.

Bacteriophages can be used successfully to fight multidrug-resistant bacteria, but although it is not difficult to find the correct virus for each specific bacterium host, the task is nevertheless not straightforward. Consequently, the use of bacteriophage particles as carriers for CRISPRs seems to be a faster and more efficient solution, although such delivery may not always be guaranteed. Recent studies show that CRISPR technology can assist in the modification of bacteriophages, making them more specific for the intended purpose.

To conclude, a deeper understanding of these new and innovative therapeutic strategies is of utmost importance. Until such new strategies have been mastered, structured, and made commercially available, it is imperative to control the use of the currently available chemical antibiotics. It is also essential that health professionals use wisely, and only as a last resort, new antibiotics that may become available in the near future, in order to prevent the emergence and spread of bacterial resistance to them.

## Author Contributions

All authors participated in writing the manuscript, specifically RL with the themes nanotechnology and CRISPRs, FF with multidrug resistance, and VB with bacteriophage technology.

## Funding

The funding for this work was provided by the São Paulo State Research Foundation [FAPESP, grants #2016/08884-3 (PneumoPhageColor project), #2016/12234-4 (TransAppIL project), #2018/05522-9 (PsaPhageKill project, BPE fellowship granted to VB), and #2017/13328-5 (Biogenic Metal Nanoparticles project)]. Support was provided by the National Council for Scientific and Technological Development (CNPq), in the form of Research Productivity (PQ) fellowships awarded to VB (grants #306113/2014-7 and #308208/2017-0) and RL (grant #303967/2015-3). Funding support was also provided by CESAM (UID/AMB/50017/2019) and FCT/MCTES.

## Conflict of Interest Statement

The authors declare that the research was conducted in the absence of any commercial or financial relationships that could be construed as a potential conflict of interest.

## References

[B1] Ahmad A. Wei Y. Syed F. Tahir K. Taj R. Khan A. U. (2016). Amphotericin B-conjugated biogenic silver nanoparticles as an innovative strategy for fungal infections. Microb. Pathog. 99, 271–281. 10.1016/j.micpath.2016.08.031 27591110

[B2] Alekshun M. N. Levy S. B. (2007). Molecular mechanisms of antibacterial multidrug resistance. Cell 128 (6), 1037–1050. 10.1016/j.cell.2007.03.004 17382878

[B3] Ali J. Rafiq Q. A. Ratcliffe E. (2018). Antimicrobial resistance mechanisms and potential synthetic treatments. Future Sci. OA. 4 (4), Fso290. 10.4155/fsoa-2017-0109 29682325 PMC5905577

[B4] Ansari M. A. Khan H. M. Khan A. A. Cameotra S. S. Saquib Q. Musarrat J. (2014). Interaction of Al2O3 nanoparticles with Escherichia coli and their cell envelope biomolecules. J. Appl. Microbiol. 116 (4), 772–783. 10.1111/jam.12423 24354999

[B5] Azam A. Ahmed A. S. Oves M. Khan M. S. Habib S. S. Memic A. (2012). Antimicrobial activity of metal oxide nanoparticles against gram-positive and gram-negative bacteria: a comparative study. Int. J. Nanomedicine 7, 6003–6009. 10.2147/IJN.S35347 23233805 PMC3519005

[B6] Balashanmugam P. Kalaichelvan P. T. (2015). Biosynthesis characterization of silver nanoparticles using Cassia roxburghii DC aqueous extract, and coated on cotton cloth for effective antibacterial activity. Int. J. Nanomedicine 10 (1), 87–97. 10.2147/IJN.S79984 26491310 PMC4599608

[B7] Balcão V. M. Barreira S. V. P. Nunes T. M. Chaud M. V. Tubino M. Vila M. M. D. C. (2014). Carbohydrate hydrogels with stabilized phage particles for bacterial biosensing: bacterium diffusion studies. *Appl* . Biochem. Biotechnol. 172, 1194–1214. 10.1007/s12010-013-0579-2 24146368

[B8] Balcão V. M. Moreira A. R. Moutinho C. G. Chaud M. V. Tubino M. Vila M. M. (2013). Structural and functional stabilization of phage particles in carbohydrate matrices for bacterial biosensing. . Technol. 53, 55–69. 10.1016/j.enzmictec.2013.03.001 23683705

[B9] Balcão V. M. Vila M. M. D. C. (2015). Structural and functional stabilization of protein entities: state-of-the-art. Adv. Drug Deliv. Rev. 93, 25–41. 10.1016/j.addr.2014.10.005 25312675

[B10] Barrangou R. (2015). The roles of CRISPR–Cas systems in adaptive immunity and beyond. Curr. Opin. Immunol. 32, 36–41. 10.1016/j.coi.2014.12.008 25574773

[B11] Beisel C. L. Gomaa A. A. Barrangou R. (2014). A CRISPR design for next-generation antimicrobials. Genome Biol. 15, 516. 10.1186/s13059-014-0516-x 25417800 PMC4282009

[B12] Bell B. G. Schellevis F. Stobberingh E. Goossens H. Pringle M. (2014). A systematic review and meta-analysis of the effects of antibiotic consumption on antibiotic resistance. Bioorg. Med. Chem. Lett. 14, 13. 10.1186/1471-2334-14-13 PMC3897982 24405683

[B13] Bikard D. Barrangou R. (2017). Using CRISPR–Cas systems as antimicrobials. Curr. Opin. Microbiol. 37, 155–160. 10.1016/j.mib.2017.08.005 28888103

[B14] Bikard D. Euler C. W. Jiang W. Nussenzweig P. M. Goldberg G. W. Duportet X. (2014). Exploiting CRISPR–Cas nucleases to produce sequence-specific antimicrobials. Nat. Biotechnol. 32 (11), 1146–1150. 10.1038/nbt.3043 25282355 PMC4317352

[B15] Boucher H. W. Talbot G. H. Bradley J. S. Edwards J. E. Gilbert D. Rice L. B. (2009). Bad bugs, no drugs: no ESKAPE! an update from the Infectious diseases society of America. Dis. Soc. Am. 48 (1), 1–12. 10.1086/595011 19035777

[B16] Boya V. N. Lovett R. Setua S. Gandhi V. Nagesh P. K. B. Khan S. (2017). Probin mucin interaction behavior of magnetic nanoparticles. J. Coll. Inter. Sci. 488, 258–268. 10.1016/j.jcis.2016.10.090 PMC5461659 27837716

[B17] Brunet L. Lyon D. Y. Hotze E. M. Alvarez P. J. Wiesner M. R. (2009). Comparative photoactivity and antibacterial properties of C60 fullerenes and titanium dioxide nanoparticles. Environ. Sci. Technol. 43 (12), 4355–4360. 10.1021/es803093t 19603646

[B18] Chan B. K. Abedon S. T. (2012). “Phage therapy pharmacology phage cocktails,” in Advances in applied microbiology, vol. 78 Eds. Laskin A. I. Sariaslani S. Gadd G. M. (San Diego: Elsevier Academic Press Inc.), 1–23. 10.1016/B978-0-12-394805-2.00001-4 22305091

[B19] Chen M. Yang Z. Wu H. Pan X. Xie X. Wu C. (2011). Antimicrobial activity and the mechanism of silver nanoparticle thermosensitive gel. Int. J. Nanomedicine. 6, 2873–2877. 10.2147/IJN.S23945 22131833 PMC3224714

[B20] Chen S. Liu H. Liang W. Hong L. Zhang B. Huang L. (2019). Insertion sequences in the CRISPR–Cas system regulate horizontal antimicrobial resistance gene transfer in. Int. J. Antimicrob. Agents 53 (2), 109–115. 10.1016/j.ijantimicag.2018.09.020 30290202

[B21] Chernousova S. Epple M. (2013). Silver as antibacterial agent: ion, nanoparticle, and metal. Angew. Chem. Int. Ed. Engl. 52 (6), 1636–1653. 10.1002/anie.201205923 23255416

[B22] Chibani-Chennoufi S. Bruttin A. Dillmann M.-L. Brüssow H. (2004). Phage–host interaction: an ecological perspective. J. Bacteriol. 186 (12), 3677–3686. 10.1128/JB.186.12.3677-3686.2004 15175280 PMC419959

[B23] Choi O. Hu Z. (2008). Size dependent and reactive oxygen species related nanosilver toxicity to nitrifying bacteria. Environ. Sci. Technol. 42 (12), 4583–4588. 10.1021/es703238h 18605590

[B24] Chwalibog A. Sawosz E. Hotowy A. Szeliga J. Mitura S. Mitura K. (2010). Visualization of interaction between inorganic nanoparticles and bacteria or fungi. Int. J. Nanomedicine. 5, 1085–1094. 10.2147/IJN.S13532 21270959 PMC3023237

[B25] Citorik R. J. Mimee M. Lu T. K. (2014). Sequence-specific antimicrobials using efficiently delivered RNA-guided nucleases. Nat. Biotechnol. 32 (11), 1141–1145. 10.1038/nbt.3011 25240928 PMC4237163

[B26] Cong S. Cao Y. Fang X. Wang Y. Liu Q. Gui H. (2016). Carbon nanotube macroelectronics for active matrix polymer-dispersed liquid crystal displays. ACS Nano. 10 (11), 10068–10074. 10.1021/acsnano.6b04951 27763766

[B27] Dakal T. C. Kumar A. Majumdar R. S. Yadav V. (2016). Mechanistic basis of antimicrobial actions of silver nanoparticles. Front. Microbiol. 7, 1831. 10.3389/fmicb.2016.01831 27899918 PMC5110546

[B28] Derbali R. M. Aoun V. Moussa G. Frei G. Tehrani S. F. Del’Orto J. C. (2019). Tailored nanocarriers for the pulmonary delivery of levofloxacin against Pseudomonas aeruginosa: a comparative study. Mol. Pharm. 16 (5), 1906–1916. 10.1021/acs.molpharmaceut.8b01256 30900903

[B29] Dizaj S. M. Lotfipour F. Barzegar-Jalali M. Zarrintan M. H. Adibkia K. (2014). Antimicrobial activity of the metals and metal oxide nanoparticles, mater. Sci. Eng. C: Mater. Biol. Appl. 44, 278–284. 10.1016/j.msec.2014.08.031 25280707

[B30] Doss J. Culbertson K. Hahn D. Camacho J. Barekzi N. (2017). A review of phage therapy against bacterial pathogens of aquatic and terrestrial organisms. Viruses 9, 50. 10.3390/v9030050 PMC5371805 28335451

[B31] Durán N. Durán M. de Jesus M. B. Seabra A. B. Fávaro W. J. Nakazato G. (2016a). Silver nanoparticles: a new view on mechanistic aspects on antimicrobial activity. Nanomedicine 12 (3), 789–799. 10.1016/j.nano.2015.11.016 26724539

[B32] Durán N. Nakazato G. Seabra A. B. (2016b). Antimicrobial activity of biogenic silver nanoparticles, and silver chloride nanoparticles: an overview and comments. Appl. Microbiol. Biotechnol. 100 (15), 6555–6570. 10.1007/s00253-016-7657-7 27289481

[B33] Durán N. Marcato P. D. De Conti R. Alves O. L. Costa F. T. M. Brocchi M. (2010). Potential use of silver nanoparticles on pathogenic bacteria, their toxicity and possible mechanisms of action. J. Braz. Chem. Soc. 21 (6), 949–959. 10.1590/S0103-50532010000600002

[B34] Eymard-Vernain E. Luche S. Rabilloud T. Lelong C. (2018). Impact of nanoparticles on the Bacillus subtilis (3610) competence. *Sci. Rep.* 8: 2978, Correction in: *Sci. Rep.* 2018 8, 6486. 10.1038/s41598-018-21402-0 PMC5813000 29445231

[B35] Falagas M. E. Kasiakou S. K. (2005). Colistin: the revival of polymyxins for the management of multidrug-resistant gram-negative bacterial infections. Infect. Dis. Soc. Am. 40 (9), 1333–1341. 10.1086/429323 15825037

[B36] Forsberg K. J. Patel S. Gibson M. K. Lauber C. L. Knight R. Fierer N. (2014). Bacterial phylogeny structures soil resistomes across habitats. Nature 509, 612–616. 10.1038/nature13377 24847883 PMC4079543

[B37] Fuente-Núñez C. Lu T. K. (2017). CRISPR–Cas9 technology: applications in genome engineering, development of sequence-specific antimicrobials, and future prospects. Integr. Biol. 9, 109–122. 10.1039/c6ib00140h 28045163

[B38] Gaspar D. P. Gaspar M. M. Eleutério C. V. Grenha A. Blanco M. Gonçalves L. M. D. (2017). Microencapsulated solid lipid nanoparticles as a hybrid platform for pulmonary antibiotic delivery. Mol. Pharm. 514 (9), 2977–2990. 10.1021/acs.molpharmaceut.7b00169 28809501

[B39] Gomaa A. A. Klumpe H. E. Luo M. L. Selle K. Barrangou R. Beisel C. L. (2014). Programmable removal of bacterial strains by use of genome-targeting CRISPR–Cas systems. MBio. 5 (1), e00928–00913. 10.1128/mBio.00928-13 PMC3903277 24473129

[B40] Gonzalez-Bello C. (2017). Antibiotic adjuvants—a strategy to unlock bacterial resistance to antibiotics. Bioorg. Med. Chem. Lett. 27 (18), 4221–4228. 10.1016/j.bmcl.2017.08.027 28827113

[B41] Grigor’eva A. Saranina I. Tikunova N. Safonov A. Timoshenko N. Rebrov A. (2013). Fine mechanisms of the interaction of silver nanoparticles with the cells of Salmonella typhimurium and staphylococcus aureus. Biometals 26 (3), 479–488. 10.1007/s10534-013-9633-3 23686387

[B42] Guilger M. Pasquoto-Stigliani T. Bilesky-José N. Grillo R. Abhilash P. C. Fraceto L. F. (2017). Biogenic silver nanoparticles based on Trichoderma harzianum: synthesis, characterization, toxicity evaluation and biological activity. Sci. Rep. 7, 44421. 10.1038/srep44421 28300141 PMC5353535

[B43] Gurunathan S. Han J. W. Dayem A. A. Eppakayala V. Kim J. H. (2012). Oxidative stress-mediated antibacterial activity of graphene oxide and reduced graphene oxide in Pseudomonas aeruginosa. Int. J. Nanomedicine 7, 5901–5914. 10.2147/IJN.S37397 23226696 PMC3514835

[B44] Habash M. B. Park A. J. Vis E. C. Harris R. J. Khursigara C. M. (2014). Synergy of silver nanoparticles and aztreonam against Pseudomonas aeruginosa PAO1 biofilms. Antimicrob. Agents Chemother. 58 (10), 5818–5830. 10.1128/AAC.03170-14 25049240 PMC4187931

[B45] Hagens S. Loessner M. J. (2010). Bacteriophage for biocontrol of foodborne pathogens: calculations and considerations. Curr. Pharm. Biotechnol. 11 (1), 58–68. 10.2174/138920110790725429 20214608

[B46] Hajipour M. J. Fromm K. M. Ashkarran A. A. Jimenez de Aberasturi D. de Larramendi I. R. Rojo T. (2012). Antibacterial properties of nanoparticles. Trends Biotechnol. 30 (10), 499–511. 10.1016/j.tibtech.2012.06.004 22884769

[B47] Hao M. Ye M. Shen Z. Hu F. Yang Y. Wu S. (2018). Porin deficiency in carbapenem-resistant enterobacter aerogenes strains. Microb. Drug Resist. 24 (9), 1–7. 10.1089/mdr.2017.0379 29653477

[B48] Harada L. K. Silva E. C. Campos W. F. Del Fiol F. S. Vila M. Dąbrowska K. (2018). Biotechnological applications of bacteriophages: state of the art. Microbiol. Res. 212–213, 38–58. 10.1016/j.micres.2018.04.007 29853167

[B49] Hatoum-Aslan A. (2018). Phage genetic engineering using CRISPR–Cas systems. Viruses 10, 335. 10.3390/v10060335 PMC6024849 29921752

[B50] He Y. Ingudam S. Reed S. Gehring A. Strobaugh T. P. Jr. Irwin P. (2016). Study on the mechanism of antibacterial action of magnesium oxide nanoparticles against foodborne pathogens. J. Nanobiotechnology 14, 54. 10.1186/s12951-016-0202-0 27349516 PMC4924328

[B51] Hemeg H. A. (2017). Nanomaterials for alternative antibacterial therapy. Int. J. Nanomedicine 12, 8211–8225. 10.2147/IJN.S132163 29184409 PMC5689025

[B52] Hogberg L. D. Heddini A. Cars O. (2010). The global need for effective antibiotics: challenges and recent advances. Trends Pharmacol. Sci. 31 (11), 509–515. 10.1016/j.tips.2010.08.002 20843562

[B53] Hong W. Gao X. Qiu P. Yang J. Qiao M. Shi H. (2017). Synthesis, construction, and evaluation of self-assembled nano-bacitracin A as an efficient antibacterial agent in vitro and in vivo. Int. J. Nanomedicine 12, 4691–4708. 10.2147/IJN.S136998 28721045 PMC5501637

[B54] Hsu P. D. Lander E. S. Zhang F. (2014). Development and applications of CRISPR–Cas9 for genome engineering. Cell 157 (6), 1262–1278. 10.1016/j.cell.2014.05.010 24906146 PMC4343198

[B55] Hullahalli K. Rodrigues M. Palmer K. L. (2017). Exploiting CRISPR–cas to manipulate enterococcus faecalis populations. Elife 6, e26664. 10.7554/eLife.26664 28644125 PMC5491264

[B56] Hullahalli K. Rodrigues M. Nguyen U. T. Palmer K. (2018). An attenuated CRISPR–Cas system in Enterococcus faecalis permits DNA acquisition. MBio. 9 (3), e00414–18. 10.1128/mBio.00414-18 PMC5930301 29717009

[B57] Hyman P. Abedon S. T. (2010). “Bacteriophage host range and bacterial resistance,” in Advances in applied microbiology, vol. 70 Eds. Laskin A. I. Sariaslani S. Gadd G. M. (San Diego: Elsevier Academic Press Inc.), 217–248. 10.1016/S0065-2164(10)70007-1 20359459

[B58] Jamil B. Imran M. (2018). Factors pivotal for designing of nanoantimicrobials: an exposition. Crit. Rev. Microbiol. 44 (1), 79–94. 10.1080/1040841X.2017.1313813 28421881

[B59] Jansen K. U. Knirsch C. Anderson A. S. (2018). The role of vaccines in preventing bacterial antimicrobial resistance. Nat. Med. 24 (1), 10–19. 10.1038/nm.4465 29315295

[B60] Jung W. K. Koo H. C. Kim K. W. Shin S. Kim S. H. Park Y. H. (2008). Antibacterial activity and mechanism of action of the silver ion in Staphylococcus aureus and. Appl. Environm. Microbiol. 74 (7), 2171–2178. 10.1128/AEM.02001-07 PMC2292600 18245232

[B61] Kalhapure R. S. Suleman N. Mocktar C. Seedat N. Govender T. (2014). Nanoengineered drug delivery systems for enhancing antibiotic therapy. J. Pharm. Sci. 104 (3), 872–905. 10.1002/jps.24298. 25546108

[B62] Kapoor G. Saigal S. Elongavan A. (2017). Action and resistance mechanisms of antibiotics: a guide for clinicians. J. Anaesthesiol. Clin. Pharmacol. 33 (3), 300–305. 10.4103/joacp.JOACP_349_15 29109626 PMC5672523

[B63] Kasithevar M. Periakaruppan P. Muthupandian S. Mohan M. (2017). Antibacterial efficacy of silver nanoparticles against multi-drug resistant clinical isolates from post-surgical wound infections. Microb. Pathog. 107, 327–334. 10.1016/j.micpath.2017.04.013 28411059

[B64] Khan S. Alam F. Azam A. Khan A. U. (2012). Gold nanoparticles enhance methylene blue-induced photodynamic therapy: a novel therapeutic approach to inhibit Candida albicans biofilm. Int. J. Nanomedicine 7, 3245–3257. 10.2147/IJN.S31219 22802686 PMC3396389

[B65] Kim S. Y. Li B. Linhardt R. J. (2017). Pathogenesis and inhibition of flaviviruses from a carbohydrate perspective. Pharmaceuticals 10, 44. 10.3390/ph10020044 PMC5490401 28471403

[B66] Kon K. Rai M. (2013). Metallic nanoparticles: mechanism of antibacterial action and influencing factors. J. Comp. Clin. Pathol. Res. 2, 160–174.

[B67] Kumar M. Curtis A. Hoskins C. (2018). Application of nanoparticle technologies in the combat against anti-microbial resistance. Pharmaceutics 10 (1), 11. 10.3390/pharmaceutics10010011 PMC5874824 29342903

[B68] Kumar T. S. Madhumathi K. (2016). Antibiotic delivery by nanobioceramics. Ther. Deliv. 7 (8), 573–588. 10.4155/tde-2016-0025 27444496

[B69] Li J. Rong K. Zhao H. Li F. Lu Z. Chen R. (2013). Highly selective antibacterial activities of silver nanoparticles against Bacillus subtilis. J. Nanosci. Nanotechnol. 13 (10), 6806–6813. 10.1166/jnn.2013.7781 24245147

[B70] Li J. Qiao Y. Zhu H. Meng F. Liu X. (2014). Existence, release, and antibacterial actions of silver nanoparticles on Ag–PIII TiO2 films with different nanotopographies. Int. J. Nanomedicine 9, 3389–3402. 10.2147/IJN.S63807 25075186 PMC4106954

[B71] Lima R. Seabra A. B. Durán N. (2012). Silver nanoparticles: a brief review of cytotoxicity and genotoxicity of chemically and biogenically synthesized nanoparticles. J. Appl. Toxicol. 32 (11), 867–879. 10.1002/jat.2780 22696476

[B72] Maura D. Debarbieux L. (2011). Bacteriophages as twenty-first century antibacterial tools for food and medicine. Appl. Microbiol. Biotechnol. 90 (3), 851–859. 10.1007/s00253-011-3227-1 21491205

[B73] Meeker D. G. Wang T. Harrington W. N. Zharov V. P. Johnson S. A. Jenkins S. V. (2018). Versatility of targeted antibiotic-loaded gold nanoconstructs for the treatment of biofilm-associated bacterial infections. Int. J. Hyperthermia 34 (2), 209–219. 10.1080/02656736.2017.1392047 29025325 PMC6095133

[B74] Mocan T. Matea C. T. Pop T. Mosteanu O. Buzoianu A. D. Suciu S. (2017). Carbon nanotubes as anti-bacterial agents. Cell. Mol. Life Sci. 74 (19), 3467–3479. 10.1007/s00018-017-2532-y 28536787 PMC11107489

[B75] Moo C. L. Yang S. K. Yusoff K. Ajat M. Thomas W. Abushelaibi A. (2019). Mechanisms of antimicrobial resistance (AMR) and alternative approaches to overcome AMR. Curr. Drug Discov. Technol. 10.2174/1570163816666190304122219 30836923

[B76] Morones J. R. Elechiguerra J. L. Camacho A. Holt K. Kouri J. B. Ramírez J. T. (2005). The bactericidal effect of silver nanoparticles. Nanotechnology 16 (10), 2346–2353. 10.1088/0957-4484/16/10/059 20818017

[B77] Moutinho C. G. Matos C. M. Teixeira J. A. Balcão V. M. (2012). Nanocarrier possibilities for functional targeting of bioactive peptides and proteins: state-of-the-art. J. Drug Target. 20, 114–141. 10.3109/1061186X.2011.628397 22023555

[B78] Mulani M. S. Kamble E. E. Kumkar S. N. Tawre M. S. Pardesi K. R. (2019). Emerging strategies to combat ESKAPE pathogens in the era of antimicrobial resistance: a review. Front. Microbiol. 10, 539. 10.3389/fmicb.2019.00539 30988669 PMC6452778

[B79] Munita J. M. Arias C. A. (2016). Mechanisms of antibiotic resistance. Microbiol. Spectr. 4 (2). 10.1128/microbiolspec.VMBF-0016-2015 PMC4888801 27227291

[B80] Oliveira Santos I. C. Albano R. M. Asensi M. D. D’Alincourt Carvalho-Assef A. P. (2018). Draft genome sequence of KPC-2-producing Pseudomonas aeruginosa recovered from a bloodstream infection sample in Brazil. J. Glob. Antimicrob. Resist. 15, 99–100. 10.1016/j.jgar.2018.08.021 30172833

[B81] Nitsch-Osuch A. Gyrczuk E. Wardyn A. Życinska K. Brydak L. (2016). Antibiotic prescription practices among children with influenza. Adv. Exp. Med. Biol. 905, 25–31. 10.1007/5584_2015_198 26801146

[B82] Pan Y.-J. Lin T.-L. Chen C.-C. Tsai Y.-T. Cheng Y.-H. Chen Y.-Y. (2017). Klebsiella phage ΦK64-1 encodes multiple depolymerases for multiple host capsular types. J. Virol. 91, e02457–02416. 10.1128/JVI.02457-16 PMC5331798 28077636

[B83] Pham T. N. Loupias P. Dassonville-Klimpt A. Sonnet P. (2019). Drug delivery systems designed to overcome antimicrobial resistance. Med. Res. Rev. 2019, 1–54. 10.1002/med.21588 31004359

[B84] Poehlsgaard J. Douthwaite S. (2005). The bacterial ribosome as a target for antibiotics. Nat. Rev. Microbiol. 3 (11), 870–881. 10.1038/nrmicro1265 16261170

[B85] Prabhu S. Poulose E. K. (2012). Silver nanoparticles: mechanism of antimicrobial action, synthesis, medical applications, and toxicity effects. Int. Nano Lett. 2, 32. 10.1186/2228-5326-2-32

[B86] Pursey E. Sünderhauf D. Gaze W. H. Westra E. R. van Houte S. (2018). CRISPR–Cas antimicrobials: challenges and future prospects. PLoS Pathog. 14 (6), e1006990. 10.1371/journal.ppat.1006990 29902258 PMC6001953

[B87] Rai M. K. Deshmukh S. D. Ingle A. P. Gade A. K. (2012). Silver nanoparticles: the powerful nanoweapon against multidrug-resistant bacteria. J. Appl. Microbiol. 112 (5), 841–852. 10.1111/j.1365-2672.2012.05253.x 22324439

[B88] Redgrave L. S. Sutton S. B. Webber M. A. Piddock L. J. V. (2014). Fluoroquinolone resistance: mechanisms, impact on bacteria, and role in evolutionary success. Trends Microbiol. 22 (8), 438–445. 10.1016/j.tim.2014.04.007 24842194

[B89] Rios A. C. Vila M. M. D. C. Lima R. Del Fiol F. S. Tubino M. Teixeira J. A. (2018). Structural and functional stabilization of bacteriophage particles within the aqueous core of a W/O/W multiple emulsion: a potential biotherapeutic system for the inhalational treatment of bacterial pneumonia. Process Biochem. 64, 177–192. 10.1016/j.procbio.2017.09.022

[B90] Rios A. C. Moutinho C. G. Pinto F. C. Del Fiol F. S. Jozala A. Chaud M. V. (2016). Alternatives to overcoming bacterial resistances: state-of-the-art. Microbiol. Res. 191, 51–80. 10.1016/j.micres.2016.04.008 27524653

[B91] Rodzinski A. Guduru R. Liang P. Hadjikhani A. Stewart T. Stimphil E. (2016). Targeted and controlled anticancer drug delivery and release with magnetoelectric nanoparticles. Sci. Rep. 6, 20867. 10.1038/srep20867 26875783 PMC4753509

[B92] Ruozi B. Veratti P. Vandelli M. A. Tombesi A. Tonelli M. Forni F. (2017). Apoferritin nanocage as streptomycin drug reservoir: technological optimization of a new drug delivery system. Int. J. Pharm. 518 (1-2), 281–288. 10.1016/j.ijpharm.2016.12.038 28017769

[B93] Salem W. Leitner D. R. Zingl F. G. Schratter G. Prassl R. Goessler W. (2015). Antibacterial activity of silver and zinc nanoparticles against Vibrio cholerae and enterotoxic Escherichia coli. Int. J. Med. Microbiol. 305 (1), 85–95. 10.1016/j.ijmm.2014.11.005 25466205 PMC4300426

[B94] Senarathna U. L. N. H. Fernando S. S. N. Gunasekara T. D. C. P. Weerasekera M. M. Hewageegana H. G. S. P. Arachchi N. D. H. (2017). Enhanced antibacterial activity of TiO2 nanoparticle surface modified with Garcinia zeylanica extract. Chem. Cent. J. 11, 7. 10.1186/s13065-017-0236-x 28123449 PMC5233605

[B95] Shaaban M. I. Shaker M. A. Mady F. M. (2017). Imipenem/cilastatin encapsulated polymeric nanoparticles for destroying carbapenem-resistant bacterial isolates. J. Nanobiotechnology 15 (1), 29. 10.1186/s12951-017-0262-9 28399890 PMC5387208

[B96] Shahverdi A. Fakhimi A. Shahverdi H. Minaian S. (2007). Synthesis and effect of silver nanoparticles on the anti-bacterial activity of different antibiotics against staphylococcus aureus and. escherichia coli. Nanomed. Nanotechnol. Biol. Med. 3, 168–171. 10.1016/j.nano.2007.02.001 17468052

[B97] Shen J. Zhou J. Chen G. Q. Xiu Z. L. (2018). Efficient genome engineering of a virulent Klebsiella bacteriophage using CRISPR–Cas9. J. Virol. 92 (17), e00534–00518. 10.1128/JVI.00534-18 PMC6096830 29899105

[B98] Siemer S. Westmeier D. Barz M. Eckrich J. Wünsch D. Seckert C. (2019). Biomolecule-corona formation confers resistance of bacteria to nanoparticle-induced. Biomaterials 192, 551–559. 10.1016/j.biomaterials.2018.11.028 30530244

[B99] Sieradzki K. Markiewicz Z. (2004). Mechanism of vancomycin resistance in methicillin resistant Staphylococcus aureus. J. Microbiol. 53 (4), 207–14. 15790069

[B100] Simlai A. Mukherjee K. Mandal A. Bhattacharya K. Samanta A. Roy A. (2016). Partial purification and characterization of an antimicrobial activity from the wood extract of mangrove plant Ceriops decandra. EXCLI. J. 15, 103–112. 27065777 10.17179/excli2015-741 PMC4822046

[B101] Sorek R. Lawrence C. M. Wiedenheft B. (2013). CRISPR-mediated adaptive immune systems in bacteria and archaea. Annu. Rev. Biochem. 82, 237–266. 10.1146/annurev-biochem-072911-172315 23495939

[B102] Summers W. C. (2012). The strange history of phage therapy. Bacteriophage 2 (2), 130–133. 10.4161/bact.20757 23050223 PMC3442826

[B103] Sunitha A. Rimal I. R. S. Sweetly G. Sornalekshmi S. Arsula R. Praseetha P. K. (2013). Evaluation of antimicrobial activity of biosynthesized iron and silver nanoparticles using the fungi Fusarium oxysporum and Actinomycetes sp. Nano. Biomed. Eng. 5 (1), 39–45. 10.5101/nbe.v5i1.p39-45

[B104] Tello A. Austin B. Telfer T. C. (2012). Selective pressure of antibiotic pollution on bacteria of importance to public health. Environ. Health Perspect. 120 (8), 1100–1106. 10.1289/ehp.1104650 22571927 PMC3440082

[B105] Tran N. Mir A. Mallik D. Sinha A. Nayar S. Webster T. J. (2010). Bactericidal effect of iron oxide nanoparticles on Staphylococcus aureus. Int. J. Nanomedicine 5, 277–283. 10.2147/IJN.S9220 20463943 PMC2865022

[B106] Van Boeckel T. P. Gandra S. Ashok A. Caudron Q. Grenfell B. T. Levin S. A. (2014). Global antibiotic consumption 2000 to 2010: an analysis of national pharmaceutical sales data. Lancet Infect. Dis. 14 (8), 742–750. 10.1016/S1473-3099(14)70780-7 25022435

[B107] Verma S. K. Jha E. Panda P. K. Das J. K. Thirumurugan A. Suar M. (2018). Molecular aspects of core–shell intrinsic defect induced enhanced antibacterial activity of ZnO nanocrystals. Nanomedicine (Lond) 13 (1), 43–68. 10.2217/nnm-2017-0237 29173091

[B108] Vikesland P. Garner E. Gupta S. Kang S. Maile-Moskowitz A. Zhu N. (2019). Differential drivers of antimicrobial resistance across the world. Acc. Chem. Res. 52 (4), 916–924. 10.1021/acs.accounts.8b00643 30848890

[B109] Wang C. Wang Y. Zhang L. Miron R. J. Liang J. Shi M. (2018). Pretreated macrophage-membrane-coated gold nanocages for precise drug delivery for treatment of bacterial infections. Adv. Mater. 30 (46), e1804023. 10.1002/adma.201804023 30285289

[B110] Wang Y. Wan J. Miron R. J. Zhao Y. Zhang Y. (2016). Antibacterial properties and mechanisms of gold–silver nanocages. Nanoscale 8 (21), 11143–11152. 10.1039/C6NR01114D 27180869

[B111] Wang Y. Wang S. Chen W. Song L. Zhang Y. Shen Z. (2018). CRISPR–Cas9 and CRISPR-assisted cytidine deaminase enable precise and efficient genome editing in Klebsiella pneumoniae. Appl. Environ. Microbiol. 84 (23), e01834–01818. 10.1128/AEM.01834-18 PMC6238054 30217854

[B112] Weitz I. S. Maoz M. Panitz D. Eichler S. Segal E. (2015). Combination of CuO nanoparticles and fluconazole: preparation, characterization, and antifungal activity against Candida albicans. J. Nanopart. Res. 17 (8), 342. 10.1007/s11051-015-3149-4

[B113] Willers C. Wentzel J. F. du Plessis L. H. Gouws C. Hamman J. H. (2017). Efflux as a mechanism of antimicrobial drug resistance in clinical relevant microorganisms: the role of efflux inhibitors. Expert Opin. Ther. Targets 21 (1), 23–36. 10.1080/14728222.2017.1265105 27892739

[B114] Wittebole X. de Roock S. Opal S. M. (2013). A historical overview of bacteriophage therapy as an alternative to antibiotics for the treatment of bacterial pathogens. Virulence 4 (8), 1–10. 10.4161/viru.25991 23973944 PMC3916379

[B115] World Health Organization (2017). Global priority list of antibiotic-resistant bacteria to guide research, discovery, and development of new antibiotics.

[B116] Wu J. Y. Kim J. J. Reddy R. Wang W. M. Graham D. Y. Kwon D. H. (2005). Tetracycline-resistant clinical Helicobacter pylori isolates with and without mutations in 16S rRNA-encoding genes. Antimicrob. Agents Chemother. 49 (2), 578–583. 10.1128/AAC.49.2.578-583.2005 15673736 PMC547221

[B117] Wu S. Li A. Zhao X. Zhang C. Yu B. Zhao N. (2019). Silica-coated gold–silver nanocages as photothermal antibacterial agents for combined anti-infective therapy. ACS. Appl. Mater. Interfaces. 11 (19), 17177–17183. 10.1021/acsami.9b01149 30997794

[B118] Xiu Z. M. Ma J. Alvarez P. J. (2011). Differential effect of common ligands and molecular oxygen on antimicrobial activity of silver nanoparticles versus silver ions. Environ. Sci. Technol. 45 (20), 9003–9008. 10.1021/es201918f 21950450

[B119] Xiu Z. M. Zhang Q. B. Puppala H. L. Colvin V. L. Alvarez P. J. (2012). Negligible particle-specific antibacterial activity of silver nanoparticles. Nano. Lett. 12 (8), 4271–4275. 10.1021/nl301934w 22765771

[B120] Yan M. Wen J. Liang M. Lu Y. Kamata M. Chen I. S. Y. (2015). Modulation of gene expression by polymer nanocapsule delivery of DNA cassettes encoding small RNAs. PLoS ONE 10 (6), e0127986. 10.1371/journal.pone.0127986 26035832 PMC4452785

[B121] Yu S. Gao X. Zhang R. Li Z. Tan Z. Su H. (2016). Synthesis and characterization of α-ZrP@CHI drug deliver system. J. Nanosci. Nanotechnol. 16 (4), 3628–3631. 10.1166/jnn.2016.11859 27451678

[B122] Yuan Y.-G. Peng Q.-L. Gurunathan S. (2017). Effects of silver nanoparticles on multiple drug-resistant strains of Staphylococcus aureus and Pseudomonas aeruginosa from mastitis-infected goats: an alternative approach for antimicrobial therapy. Int. J. Mol. Sci. 18, 569. 10.3390/ijms18030569 PMC5372585 28272303

[B123] Zakharova O. V. Godymchuk A. Y. Gusev A. A. Gulchenko S. I. Vasyukova I. A. Kuznetsov D. V. (2015). Considerable variation of antibacterial activity of Cu nanoparticles suspensions depending on the storage time, dispersive medium, and particle sizes. BioMed Research International 2015, Article ID 412530, 11. 10.1155/2015/412530 26339611 PMC4538334

[B124] Zaidi S. Misba L. Khan A. U. (2017). Nano-therapeutics: a revolution in infection control in post antibiotic era. Nanomedicine 13 (7), 2281–2301. 10.1016/j.nano.2017.06.015 28673854

